# Association between Vitamin B_12_ Levels and Colon Cancer Survival: A Global Network Study

**DOI:** 10.1158/2767-9764.CRC-25-0557

**Published:** 2026-02-11

**Authors:** Bruce Chang-Gu, George Golovko, Anthony P. D’Andrea, Kamil Khanipov

**Affiliations:** 1 MD-PhD Combined Degree Program, University of Texas Medical Branch John Sealy School of Medicine, Galveston, Texas.; 2 Department of Pharmacology and Toxicology, University of Texas Medical Branch, Galveston, Texas.; 3 Department of Surgery, University of Texas Medical Branch, Galveston, Texas.

## Abstract

Recent studies have reported a link between elevated B_12_ levels and increased incidence of malignancies. In this study, we compare the association between B_12_ levels and adverse outcomes in colon cancer, including metastases and mortality. A globally federated multi-institutional database identified patients with colon cancer who had at least one B_12_ measurement within 1 year of diagnosis. Patients were stratified by B_12_ status (normal: 300–1,000, elevated: >1,000, and low: <300 pg/mL). Median overall survival (mOS) was compared via log-rank test and Cox proportional hazards models. The incidence of cancer metastases and elevated liver enzymes within 1 year of diagnosis was analyzed and compared using risk ratios. Further gene expression analysis of 283 colon cancer samples from The Cancer Genome Atlas (TCGA) and 304 normal colon tissues from the Genotype-Tissue Expression (GTEx) project was performed. A total of 37,106 patients were identified by TriNetX. High B_12_ (*n* = 6,523; mOS = 59.4 months) was associated with a decreased mOS compared with normal (*n* = 23,965; mOS = 129.8 months) and low (*n* = 5,167; mOS = 137.3 months) B_12_ patients. Patients with elevated B_12_ had higher metastatic involvement and tumor stage at diagnosis and a higher 1-year incidence of all metastases, liver, lung, and peritoneal metastases [risk ratio with 95% confidence intervals of 1.30 (1.24–1.36), 1.56 (1.45–1.68), 1.43 (1.27–1.61), 1.39 (1.24–1.55), respectively]. Gene expression analysis of TCGA and GTEx identified the expression of the B_12_-dependent methionine synthase (MTR) to be elevated in colon cancer tissue, and increased MTR expression was associated with decreased colon cancer survival (65.8 months vs. undefined). These findings identify the potential clinical relevance of B_12_ as a biomarker for colon cancer metastases and survival.

**Significance:**

Elevated B_12_ has been reported to be associated with increased cancer incidence; however, its role as a prognostic marker in colon cancer is unclear. In this multi-institutional study investigating clinical outcomes of patients with colon cancer, we demonstrate elevated B_12_ as a potential biomarker for colon cancer metastases and survival.

## Introduction

Colorectal cancer is the third most diagnosed malignancy in new cancer cases and has the second-highest cancer mortality both globally and in the United States (1–3). Localization in the colon represents the majority of new cases (61%) and mortality (61%) in patients with colorectal cancer (1). Progress in the diagnosis and treatment of colon cancer has improved the 5-year survival rates over the past three decades, with the current 5-year survival rate at 65% (4); however, colorectal cancer remains the second most deadly cancer, causing an estimated 904,000 deaths worldwide in 2022 (1, 4).

Homeostatic control of vitamin B_12_ (cobalamin) is regulated closely through the storage of B_12_ in the liver, and deficiency of this vitamin is most commonly due to either decreased dietary intake or impaired gastrointestinal absorption (5). Clinically, B_12_ deficiency manifests as anemia and/or otherwise unexplained neuropsychiatric behaviors (5). Elevated circulating B_12_ has been associated with liver cell damage, and elevated systemic B_12_ levels are commonly found in hepatic malignancies (6). The liver is the most common site for the spread of colorectal cancer, and approximately 22% of all new colorectal cancer cases have a distal metastatic site at the time of diagnosis (7). Although advancements in treatment have improved clinical outcomes for patients with metastatic colon cancer (7, 8), biomarkers for the early detection of metastatic spread of colorectal cancer remain important.

Metabolically, B_12_ is a necessary vitamin that is highly active in the liver and plays a role in both the methylation of biomolecules and the linking of the methionine and folate-dependent DNA synthesis cycles (9, 10). B_12_ is an important cofactor for methionine synthase (MTR), which facilitates the transfer of methyl groups from methyl-tetrahydrofolate to homocysteine for the generation of methionine in one-carbon metabolism (9–11). Due to its role in one-carbon metabolism and DNA synthesis, B_12_ is essential in the proliferation of rapidly dividing cells and may be targeted to inhibit cancer growth (10, 12, 13). Furthermore, there is growing evidence that increased serum and plasma B_12_ levels may be associated with cancer incidence (14–17) and mortality (16–18). Although the association between B_12_ intake and cancer incidence/survival remains controversial (6, 19–23), epidemiologic studies have reported chronic elevated B_12_ to be linked to an increased incidence of lung (24, 25) and colorectal (26) cancer.

Based on previous epidemiologic reports linking supraphysiologic B_12_ levels with colon cancer incidence, we hypothesized that B_12_ status may be associated with colon cancer survival and colon cancer metastasis. Using TriNetX, a large global federated clinical database, we provide the largest analysis of the association between B_12_ levels and colon cancer survival and metastatic outcomes. We further supplemented our TriNetX findings with gene expression data of colon cancer samples from The Cancer Genome Atlas (TCGA) as well as normal colon tissue from the Genotype-Tissue Expression (GTEx) datasets.

## Materials and Methods

### TriNetX database and patient cohort

TriNetX (RRID:SCR_022760) is a global federated health research network providing access to deidentified electronic medical records (diagnoses, procedures, medications, laboratory values, genomic information) across large healthcare organizations (HCO). This analysis was conducted using the Research Network encompassing data from 108 HCOs worldwide. Criteria for inclusion were as follows: 18 years of age or older at the time of colon cancer diagnosis [International Classification of Diseases, 10th Revision, Clinical Modification (ICD-10-CM) code C18], received at least one vitamin B_12_ lab result (LOINC: 16695-9 and LOINC: 2132-9) on the day of or within 1 year following the initial colon cancer diagnosis (ICD-10-CM code C18).

### Classification and criteria for B_12_ cohorts in TriNetX

Patient B_12_ status was determined by B_12_ lab results. Clinical cutoffs for B_12_ deficiency have previously been defined as B_12_ measurements <200 pg/mL, with values between 200 and 300 considered borderline deficient (27). Thus, low B_12_ patients in the TriNetX database were defined as having no measurement exceeding 300 pg/mL and at least one <300 pg/mL measurement within 1 year following the initial colon cancer diagnosis. We defined high B_12_ as patients with at least one measurement of >1,000 pg/mL within 1 year following initial diagnosis and who never received a B_12_ test <500 pg/mL within the same time window. Similar cutoffs have been established in a previous epidemiologic study evaluating B_12_ elevation and cancer incidence (28). Normal B_12_ was defined as patients with at least one measurement between 300 and 1,000 pg/mL and no measurement of B_12_ <300 or >1,000 pg/mL within the same period. A subset of the identified cohort with available oncologic data, including tumor–node–metastasis (TNM) characteristics and tumor staging, was also identified. Patients must have reported TNM and tumor stage data within 1 day of initial colon cancer diagnosis.

To control for potential confounders between each patient cohort, propensity score matching (PSM) for demographic characteristics and baseline medical history associated with colon cancer was performed. TriNetX performs 1:1 PSM using a logistic regression model and nearest neighbor technique with a caliper of 0.1 pooled standard deviations of aggregate propensity scores. PSM covariates included demographics (sex, age, race, and ethnicity), as well as any reported symptoms of B_12_ deficiency [neutropenia (D70), pancytopenia (D61.81), delirium due to known physiologic condition (F05)], folate levels (LOINC: 2282-2, 2284-8), surgical procedures associated with colon cancer (CPT: 45385, 1007455, 44160), prior metastatic disease diagnosis [lymph nodes (C77), respiratory and digestive organs (C78), or unspecified (C79)], radiotherapy (CPT: 1010843), common chemotherapy-related drugs [fluorouracil (RxNorm:4492), oxaliplatin (32592), leucovorin (6313), and capecitabine (194000)], and B_12_ and folic acid supplementation (ATC: B03B) within 1 year of the index event. Additional baseline characteristics of B_12_-associated medical conditions [pernicious anemia (D51.0), gastritis and duodenitis (K29), Crohn’s disease (K50), celiac disease (K90.0)] and procedures [total gastrectomy (CPT: 1007359), partial gastrectomy (1007363), bariatric surgery (1007385)] performed within 1 year of the index event were also collected but not included in the PSM criteria.

### Outcome analysis

Cohorts were divided by B_12_ status, and median overall survival (mOS) was defined as the time from diagnosis to death, with no restriction on follow-up duration in both unmatched and PSM cohorts. The incidence of metastases and elevated liver enzyme markers was evaluated in PSM cohorts within 1 year of the initial colon cancer diagnosis. Metastatic outcomes were determined by ICD-10 code, including all metastases (C77-79), liver (C78.7), lung (C78.0), and peritoneum (C78.6) metastases. Liver enzyme markers evaluated were alkaline phosphatase (AlkPhos), aspartate aminotransferase (AST), alanine aminotransferase (ALT), and bilirubin with cutoffs of >120 U/L, >40 U/L, >55 U/L, and >1.2 mg/dL, respectively.

A multivariable Cox proportional hazards model was performed using the TriNetX Analytics platform. Covariates were derived from demographic characteristics and all diagnoses, procedures, oncologic treatments, and lab values recorded within 1 year prior to the initial colon cancer diagnosis. Covariates included demographic factors (age at index, sex, race, ethnicity), cancer metastasis diagnoses (C77-79), relevant procedures, oncologic treatments, and serum folate values. Hazard ratios (HR) with 95% confidence intervals (CI) were generated by the TriNetX platform.

### Gene expression analysis

Gene expression and clinical data of patients with colon adenocarcinoma (TCGA-COAD) from TCGA (http://cancergenome.nih.gov/) and noncancer patients from GTEx were accessed through the UCSC Xena platform (https://xenabrowser.net; RRID:SCR_018938; refs. 27, 29, 30). For Kaplan–Meier analysis, gene expression data of 11 genes involved in methionine and folate metabolism were obtained from the UCSC Xena platform as log_2_(x + 1) as “IlluminaHiSeq.” Patients with colon cancer were split into high and low expression groups based on median gene expression, and patient outcomes were investigated using Kaplan–Meier and HR analysis. For MTR gene expression level comparisons across sample types, log_2_(x + 1) transformed gene expression data were obtained as “RSEM expected_count (DESeq2 standardized)” from the UCSC Xena platform. In total, 304 GTEx normal colon samples, 41 TCGA-COAD normal adjacent colon tissue samples, and 283 TCGA-COAD primary tumor samples were used for this study.

### Statistical analysis

Data gathering and statistical analysis of electronic health records were performed with TriNetX Analytics. Independent *t* tests were used to evaluate differences in continuous variables between cohorts, whereas *χ*^2^ tests were used for the analysis of categorical variables. Statistical significance was set at *P* < 0.05. Overall survival outcomes of cohorts were investigated using Kaplan–Meier analysis, and the difference between groups was tested using the log-rank test. Cox regression analysis was performed to quantify HRs with 95% CI. The one-year incidence of metastases and elevated liver enzyme markers were compared by risk ratios, and statistical significance was assessed using the risk difference provided by TriNetX. Statistical significance in multivariate analysis was determined using *P* values generated by the TriNetX Analytics platform. Final data acquisition and statistical analysis were performed on December 03, 2025.

All statistical analyses of gene expression data were performed using GraphPad Prism version 10.0.0 (RRID:SCR_002798). Survival distribution was evaluated by Kaplan–Meier analysis, and statistical significance was determined by both the log-rank test and quantified with HRs (95% CI). Normalized gene expression values from TCGA and GTEx were obtained from the UCSC-Xena platform, and ANOVA with *post hoc* Tukey comparison test determined the statistical significance of the difference in gene expression between samples. *P* < 0.05 was considered statistically significant.

## Results

### Identification of cohorts on TriNetX

**Figure 1.**
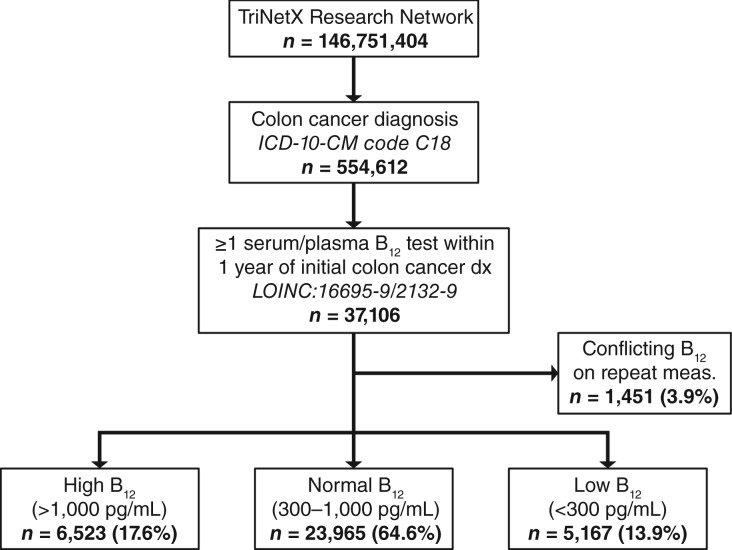
Cohort selection criteria. A total of 37,106 patients with colon cancer and at least one B_12_ measurement within 1 year of initial diagnosis were identified on the Research Network database on TriNetX by ICD-10 and LOINC codes. Patients were further stratified by B_12_ status into high (*n* = 6,523; >1,000 pg/mL), normal (*n* = 23,965; 300–1,000 pg/mL), or low (*n* = 5,167; <300 pg/mL) B_12_. Patients with conflicting repeated B_12_ measurements were excluded (*n* = 1,451).

A total of 37,106 patients with colon cancer who received B_12_ measurements within 1 year of initial colon cancer diagnosis were identified in the TriNetX database and met inclusion criteria. We report that among all patients with colon cancer identified, 64.6% (*n* = 23,965) had normal B_12_ levels, 17.6% (*n* = 6,523) had elevated B_12_ levels, and 13.9% (*n* = 5,167) had low B_12_ levels (Fig. 1). A total of 3.9% (*n* = 1,451) of patients had conflicting B_12_ measurements within this time frame and were excluded from analysis. Kaplan–Meier analysis was performed for all three cohorts.

### Elevated B_12_ levels are associated with decreased colon cancer survival

**Figure 2.**
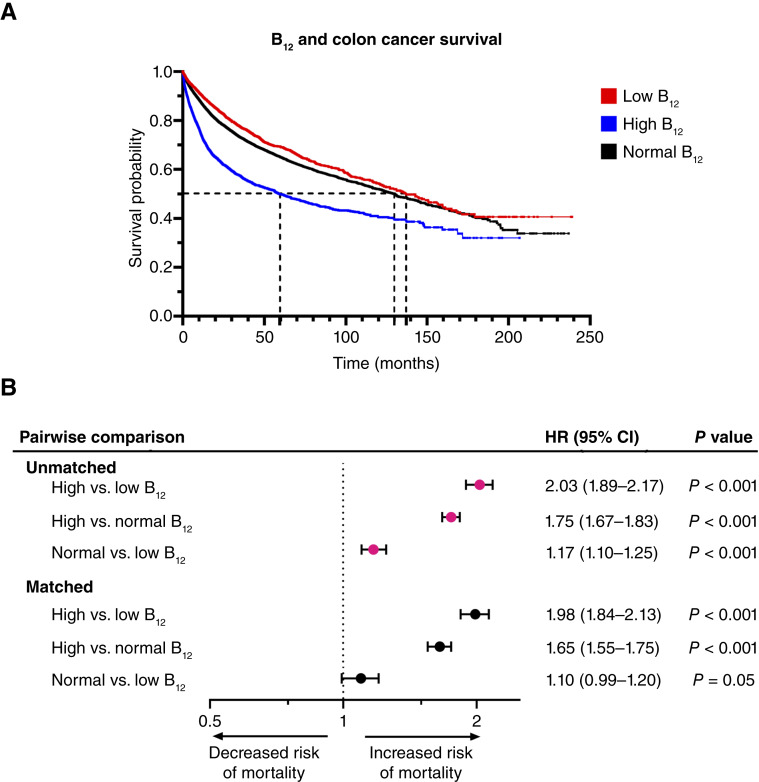
B_12_ status and colon cancer survival. **A,** Kaplan–Meier analysis of B_12_ status in unmatched patients with colon cancer and mortality. Patients with colon cancer and elevated B_12_ measurements had a decreased mOS (mOS = 59.4 months) compared with patients with normal (mOS = 129.8 months) and low B_12_ (mOS = 137.3 months). **B,** Forest plot summarizing pairwise comparisons with HR and 95% CI. Statistical significance was determined by the log-rank test.

Kaplan–Meier analysis and Cox proportional hazards model analysis were performed before and after PSM across B_12_ cohorts (Supplementary Tables S1–S3; Supplementary Fig. S1A–S1C). Before PSM, patients with colon cancer and elevated B_12_ had the lowest mOS (59.4 months) compared with patients with normal (129.8 months) and low B_12_ (137.3 months; Fig. 2A and B). After PSM, high B_12_ levels were associated with a decreased mOS compared with matched low B_12_ (*n* = 4,982 in both cohorts; mOS 64 vs. 142.9 months; *P* < 0.001; HR = 1.98; 95% CI, 1.84–2.13) and normal B_12_ patients (*n* = 6,436 in each cohort; mOS 59.4 vs. 117.8 months; *P* < 0.001; HR = 1.65; 95% CI, 1.55–1.75; Fig. 2B; Supplementary Fig. S1). After PSM, low B_12_ patients had similar mOS compared with normal B_12_ patients (Fig. 2A and B; Supplementary Fig. S1). Conditions that may be associated with B_12_ absorption, including malabsorptive conditions, pernicious anemia, and gastrectomy, were considered; however, we did not include these variates in PSM to avoid skewing cohorts to contain maladaptive B_12_ conditions. However, we do provide baseline characteristics of these conditions across unmatched cohorts (Supplementary Table S4).

Additional multivariable Cox proportional hazards analysis was performed in patients with high and normal B_12_ values to investigate other factors that may affect patient mortality (Table 1). On multivariate analyses, demographic characteristics associated with increased mortality were higher age at index (HR per year 1.02; 95% CI, 1.01–1.02; *P* value < 0.001), male sex (HR, 1.30; 95% CI, 1.25–1.36; *P* < 0.001), and being Black or African American (HR, 1.17; 95% CI, 1.07–1.29; *P* = 0.001). Across medications within 1 year before colon cancer diagnosis, prior fluorouracil use (HR, 1.28; 95% CI, 1.08–1.53; *P* = 0.01) and B_12_ and folic acid supplementation (HR, 1.13; 95% CI, 1.07–1.21; *P* < 0.001) were associated with increased mortality. Prior metastatic diagnoses to lymph nodes (HR, 1.37; 95% CI, 1.27–1.48; *P* < 0.001), respiratory and digestive organs (HR, 2.45; 95% CI, 2.31–2.59; *P* < 0.001), and unspecified other sites (HR, 1.54; 95% CI, 1.43–1.67; *P* < 0.001) led to the largest observed HRs. Conditions that decreased mortality were folate levels [HR per ng/mL 0.99 (0.98–0.99); *P* < 0.001] and colonoscopy, flexible with removal (HR, 0.71; 95% CI, 0.65–0.77; *P* < 0.001).

**Table 1.** Multivariable Cox proportional hazards model for overall survival in patients with colon cancer and high and normal B_12_ values (*n* = 30,639).

Patient characteristic	HR	95% CI	*P* value
High B_12_ (>1,000 pg/mL) vs. normal B_12_	1.63	1.56–1.72	**<0.001**
Demographic	​	​	​
Male	1.30	1.25–1.36	**<0.001**
Age at index	1.02	1.01–1.02	**<0.001**
Hispanic or Latino	0.98	0.88–1.09	0.70
Asian	0.92	0.80–1.05	0.21
Black/African American	1.17	1.07–1.29	**0.001**
White	1.06	0.98–1.16	0.15
Procedures	​	​	​
Colonoscopy, flexible with removal	0.71	0.65–0.77	**<0.001**
Colectomy, partial	1.07	0.92–1.26	0.38
Colectomy, partial with ostomy	0.99	0.80–1.22	0.91
Labs	​	​	​
Folate levels (continuous)	0.99	0.98–0.99	**<0.001**
Treatment	​	​	​
Radiation treatment	1.10	0.98–1.23	0.12
Oxaliplatin	1.03	0.85–1.25	0.77
Fluorouracil	1.28	1.08–1.53	**0.01**
Capecitabine	1.02	0.87–1.19	0.81
Leucovorin	1.12	0.89–1.40	0.33
Metastatic diagnosis	​	​	​
Secondary neoplasm of lymph nodes	1.37	1.27–1.48	**<0.001**
Respiratory and digestive organs	2.45	2.31–2.59	**<0.001**
Other sites	1.54	1.43–1.67	**<0.001**
Supplementation	​	​	​
B_12_ and folic acid	1.13	1.07–1.21	**<0.001**

Bolded values represent statistical significance (P < 0.05).

### Elevated B_12_ is associated with increased cancer stage and metastasis

The significant associations we found between elevated B_12_ and colon cancer mortality suggest B_12_ may be associated with colon cancer stage and/or metastasis. Although oncologic data are limited in the TriNetX database, of the identified patients with colon cancer, 1,715 patients (high B_12_, *n* = 355; normal B_12_, *n* = 1,175; low B_12_, *n* = 185) have cancer staging and TNM categorical status recorded within 1 day of the initial colon cancer diagnosis (Supplementary Table S5). High B_12_ patients had a higher percentage of stage 4 tumors than normal B_12_ (54% vs. 36%, *P* < 0.001) and low B_12_ patients (54% vs. 28%, *P* < 0.001). High B_12_ colon cancer patients had a higher percentage of T4 tumors than normal B_12_ (32% vs. 26%, *P* = 0.049) and low B_12_ (32% vs. 23%, *P* = 0.04). Additionally, patients with high B_12_ had higher metastasis at diagnosis than normal B_12_ (53% vs. 35%, *P* < 0.001) and low B_12_ (53% vs. 26%, *P* < 0.001) patients. Patients had similar node scoring regardless of B_12_ status. These results suggest that patients with high B_12_ have an increased metastatic burden at diagnosis.

Given the liver’s role in both B_12_ homeostasis and as a common site for colon cancer metastasis, we next examined metastases and liver function across cohorts. Using PSM cohorts that accounted for prior metastatic disease (ICD-10 codes C77–C79), high B_12_ patients had a higher incidence of metastases within 1 year after diagnosis compared with matched normal B_12_ patients: all metastases (41.7% vs. 32.1%), liver (23.1% vs. 14.8%), lung (9.6% vs. 6.7%), and peritoneum (10.5% vs. 7.6%; Table 2). We further evaluated liver dysfunction by assessing the incidence of elevated liver enzymes within 1 year. High B_12_ patients had higher rates of AlkPhos (>120 U/L, 57.4% vs. 40.8%), AST (>40 U/L, 52.9% vs. 38.6%), ALT (>55 U/L, 32.9% vs. 23.9%), and bilirubin (>1.2 mg/dL, 31.6% vs. 21%) relative to normal B_12_ patients (Table 2).

**Table 2.** Outcome analyses comparing rates of metastases and elevated liver enzyme markers within 1 year of initial colon cancer diagnosis in matched high and normal B_12_ patients with colon cancer.

​	High B_12_ (*n* = 6,436)	Normal B_12_ (*n* = 6,436)	Risk ratio	*P* values
Metastases (C77-79)	41.7%	32.1%	1.30 (1.24–1.36)	**<0.001**
Liver (C78.7)	23.1%	14.8%	1.56 (1.45–1.68)	**<0.001**
Lung (C78.0)	9.6%	6.7%	1.43 (1.27–1.61)	**<0.001**
Peritoneum (C78.6)	10.5%	7.6%	1.39 (1.24–1.55)	**<0.001**
Liver enzyme markers	​	​	​	​
AlkPhos (>120 U/L)	57.4%	40.8%	1.41 (1.36–1.46)	**<0.001**
AST (>40 U/L)	52.9%	38.6%	1.37 (1.32–1.43)	**<0.001**
ALT (>55 U/L)	32.9%	23.9%	1.38 (1.30–1.46)	**<0.001**
Bilirubin (>1.2 mg/dL)	31.6%	21%	1.51 (1.42–1.60)	**<0.001**

Bolded values represent statistical significance (P < 0.05).

### Gene expression levels of the B_12_-dependent MTR linked to worsened cancer survival among patients with colon cancer

**Figure 3.**
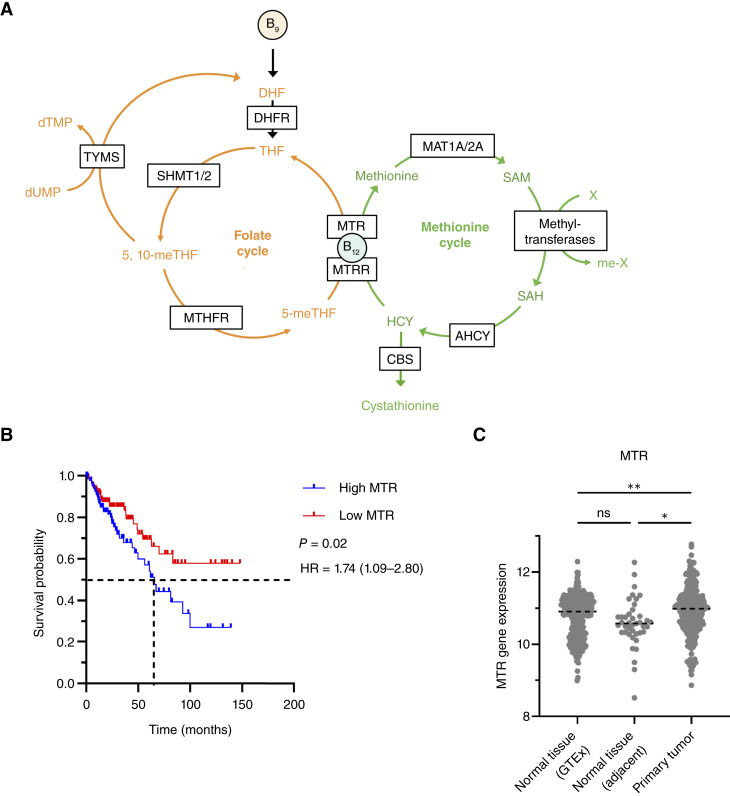
Altered methionine and folate metabolism in colon cancer. **A,** B_12_ plays a role in both the folate and methionine cycles. Key metabolites are listed along with boxed enzymes. **B,** Patients with colon cancer who have high tissue expression of MTR (*n* = 141) had a shorter overall survival rate than patients with low MTR expression (*n* = 142). The mOS of patients with high MTR expression is 66.8 months and is undefined in patients with low MTR expression due to greater than 50% patient survival at the latest follow-up. **C,** Primary colon cancer samples (*n* = 283) had higher MTR expression than normal adjacent tissue samples (*n* = 41) and normal colon tissue (*n* = 304) from noncancer patients from the GTEx database. HRs with 95% CI were determined using the Mantel–Haenszel model, and statistical significance was determined by the log-rank test and ANOVA with *post hoc* Tukey test. *, *P* < 0.05; **, *P* < 0.01. Abbreviations for enzymes are as follows: AHCY, adenosylhomocysteinase; CBS, cystathionine beta-synthase; DHFR, dihydrofolate reductase; MAT1A/2A, methionine adenosyltransferase 1A/2A; MTHFR, methylenetetrahydrofolate reductase; MTRR, methionine synthase reductase; SHMT1/2, serine hydroxymethyltransferase 1/2; TYMS, thymidylate synthase. Abbreviations for metabolites are as follows: 5,10-meTHF, 5,10-methylenetetrahydrofolate; 5-meTHF, 5-methyltetrahydrofolate; DHF, dihydrofolate; dTMP, deoxythymidine monophosphate; dUMP, deoxyuridine monophosphate; HCY, homocysteine; SAH, S-adenosylhomocysteine; SAM, S-adenosylmethionine; THF, tetrahydrofolate.

**Figure 4.**
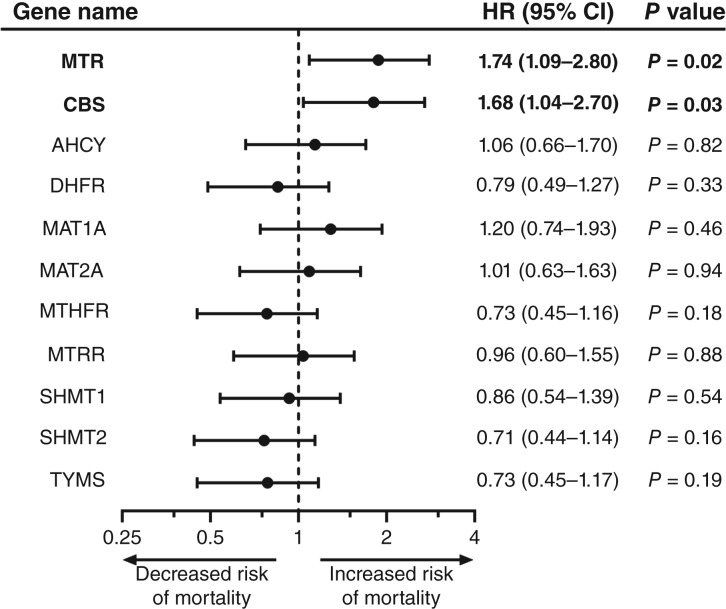
Differential expression of genes involved in methionine and folate metabolism in colon cancer. Key genes involved in methionine and folate metabolism were identified, and patients with colon cancer from the TCGA database were stratified into cohorts based on median expression levels of each gene. Kaplan–Meier survival analyses were performed, and HRs with 95% CI were determined, with HR greater than 1 meaning increased gene expression associated with increased risk of mortality. Genes in bold indicate those in which elevated expression was significantly associated with increased mortality.

To further probe B_12_’s role in colon cancer survival, we performed survival analyses of 11 genes involved in both the methionine and folate cycles (Fig. 3A) based on RNA sequencing data from TCGA (283 colon cancer tissue samples and 41 normal adjacent colon tissues from patients with colon cancer) and the GTEx dataset (304 normal colon tissues from noncancer patients). In four of the colon cancer samples, there was no detected expression of MAT1A, one of the enzymes involved in the methionine cycle (Fig. 3A; Supplementary Fig. S2); these samples were excluded from the Kaplan–Meier analysis. Of the genes investigated, we discovered that increased expression of the B_12_-dependent MTR (*n* = 141) was linked to decreased mOS compared with patients with low MTR (*n* = 142; mOS 65.8 months vs. undefined, *P* = 0.02; HR = 1.74; 95% CI, 1.09–2.80) expression (Fig. 3B). We additionally found that MTR expression was significantly higher in colon cancer primary tumors compared with normal adjacent colon tissue from biopsy (*P* = 0.03) and normal colon tissue from the GTEx database (*P* = 0.004; Fig. 3C). Of the remaining probed genes, only elevated cystathionine beta-synthase expression (*n* = 141) was associated with a decrease in survival compared with low cystathionine beta-synthase expressing patients (*n* = 142; mOS 67.3 vs. 92.7 months; *P* = 0.03; HR = 1.68; 95% CI, 1.04–2.70; Fig. 4; Supplementary Fig. S2).

## Discussion

Vitamin irregularities are common among patients with cancer; however, the exact relationship between B_12_ and malignancies remains unclear. In this large, multicenter retrospective study, we report that elevated serum/plasma B_12_ levels are associated with significantly decreased mOS in patients with colon cancer. This association remained significant after PSM for confounding variables. Notably, elevated B_12_ levels were also linked to higher rates of metastatic disease both at diagnosis and within 1 year of diagnosis. We further performed gene expression studies in the TCGA and GTEx databases and found the B_12_-dependent MTR to be highly expressed in colon cancer and associated with decreased colon cancer survival. Together, these results suggest that elevated B_12_ levels in patients with colon cancer are a clinically significant finding, and the metabolism of B_12_ may play a role in colon cancer biology.

One of the earliest reports of B_12_ abnormalities and cancer survival was by Carmel and Eisenberg, in which they reported that elevated B_12_ levels were associated with decreased cancer survival among 150 patients with solid tumors (17). Additionally, a more extensive study by Arendt and colleagues (14, 16) of patients with cancer within the Danish medical registry reported that elevated B_12_ levels were associated with increased cancer mortality. These studies have been limited to single-institution studies or homogeneous patient populations from a single country (14–17). The current study is the first multinational investigation that demonstrates supraphysiologic B_12_ levels are associated with decreased colon cancer survival. We used PSM to balance cohorts based on demographic characteristics (sex, age at index, race/ethnicity), symptoms of B_12_ deficiency, chemotherapy and radiotherapy treatment, surgical procedures related to colon cancer, and folate levels.

We report that elevated serum/plasma B_12_ levels were associated with significantly decreased colon cancer survival, an association that persisted after PSM. Additional multivariate analysis using Cox proportional hazards models of patients with colon cancer with high and normal B_12_ levels established that adverse prognostic factors, including older age at diagnosis, male sex, Black/African American ethnicity, and prior diagnosis of metastatic disease, were strongly associated with mortality. These findings support elevated B_12_ as a marker of poor prognosis but not necessarily as an independent prognostic factor. B_12_ is regulated by liver function, and abnormalities in B_12_ are associated with malabsorptive conditions, pernicious anemia, and gastrectomy procedures. These conditions are rare and did not constitute a significant portion of any of our cohorts in this study. Despite the potential adverse consequences of B_12_ deficiency, we found that patients with colon cancer and low B_12_ levels had similar survival outcomes with normal B_12_ patients. Patients with colon cancer and high B_12_ levels had an mOS of 59.4 months (5 years), which is over 5 years lower than that of patients with normal and low B_12_ levels.

Among patients with colon cancer and available oncologic data, we found that patients with elevated B_12_ had a higher stage tumor and were more likely to have metastatic involvement at diagnosis than their normal B_12_ counterparts. They also exhibited an increased 1-year incidence of all metastases, particularly to the liver, as well as elevated liver enzymes compared with normal and low B_12_ patients. Metastatic spread to the liver is the most common site for colon cancer, and a significant number of new patients with colon cancer have distal metastases at the time of diagnosis (7). Previously, Lin and colleagues (6) reported that elevated B_12_ levels in patients with hepatocellular carcinoma were not associated with excess dietary vitamin intake but rather with liver dysfunction and that serum B_12_ levels were a predictive prognostic factor of survival in patients with hepatocellular carcinoma. Similarly, we find that elevated B_12_ in patients with colon cancer is associated with an increased incidence of all metastases, including liver metastasis, and elevated liver enzymes (ALT, AST, AlkPhos, and bilirubin). Thus, hepatic involvement may be a contributing factor to abnormal serum B_12_ values and the observed decreased survival in some patients with colon cancer.

Although B_12_ is often a marker of liver involvement, our gene expression findings suggest it may also play a role in tumor metabolism. Given the important role of B_12_ in DNA synthesis and cell division, and the potential role B_12_ may play in cancer biology, we next investigated gene expression data of B_12_-related enzymes in the TCGA and GTEx databases. Notably, elevated expression of the B_12_-dependent MTR was one of only two genes within the folate and methionine cycles that correlated with worsened overall survival in patients with colon cancer. MTR is a B_12_-dependent enzyme that connects the folate and methionine cycles, allowing for both DNA synthesis and one-carbon metabolism to occur. Previous studies have shown MTR to be essential for tumor formation in mouse models (12), and our results suggest that colon cancer tissue may have an increased metabolic demand for B_12_ and methionine driven by MTR; however, further mechanistic studies will be required.

Interestingly, the second gene we identified was cystathionine beta-synthase, which catalyzes the first step of the transsulfuration pathway and connects the methionine and cysteine metabolic pathways by synthesizing cystathionine from homocysteine, leading to the generation of endogenous hydrogen sulfide (31, 32). Previous immunoblotting studies have reported cystathionine beta-synthase protein to be highly expressed in colon cancer tissue compared with normal surrounding mucosa and to play a role in carcinogenesis (33, 34). Based on functional studies silencing cystathionine beta-synthase gene expression, it is thought that cystathionine beta-synthase–generated hydrogen sulfide promotes tumor growth by promoting cell proliferation and angiogenesis (31–33). These reports support our observation that elevated cystathionine beta-synthase gene expression in colon cancer tissue is associated with decreased colon cancer survival.

Our study is limited due to the retrospective nature of our analysis, as well as the limited oncologic data available on TriNetX. Given that our analysis is observational, we cannot determine the primary cause of high B_12_ levels observed in some patients with colon cancer and whether it is driven by cancer infiltration to the liver and/or dietary excess. Future work will be required to determine the causal relationship between B_12_ homeostasis and colon cancer progression. Additionally, TriNetX does not support survival modeling with continuous B_12_ levels, limiting the evaluation of B_12_ level–response effects. The database also does not include sufficient information to reliably calculate disease-free survival; however, we report the incidence of metastatic diagnoses within 1 year of the initial colon cancer diagnosis as a proxy for metastatic potential. Despite these limitations, this is the first large-scale multinational analysis demonstrating a clinically significant association between B_12_ and colon cancer survival. Although chemotherapy for colon cancer typically targets the folate cycle (capecitabine and 5-fluorouracil), there are no FDA-approved drugs targeting the methionine cycle despite the well-documented increased demand for methionine in cancer (12, 35–37). Our findings suggest that elevated B_12_ levels in colon cancer are strongly associated with decreased survival and may be a biomarker for adverse outcomes, including colon cancer metastasis and liver dysfunction.

## Supplementary Material

Supplemental Figure S1 Kaplan-Meier curves based on B12 status after propensity score matching.

Supplemental Figure S2 Gene expression levels of enzymes involved in methionine and folate cycle and colon cancer survival.

Supplemental Table S1 Characteristics of High and Low B12 Colon Cancer Patients Before and After Propensity Score Matching.

Supplemental Table S2 Characteristics of High and Normal B12 Colon Cancer Patients Before and After Propensity Score Matching.

Supplemental Table S3 Characteristics of Normal and Low B12 Colon Cancer Patients Before and After Propensity Score Matching.

Supplemental Table S4 Background characteristics of maladaptive B12 conditions across cohorts.

Supplemental Table S5 Cancer staging data from colon cancer patients at index event with high, normal, and low B12 values.

## Data Availability

Patient data analyzed in this study are available from TriNetX, LLC. Restrictions apply to the availability of these data, which were used under license for this study. Data are available from the authors upon reasonable request with the permission of TriNetX, LLC. Gene expression and clinical data analyzed in this study from TCGA (http://cancergenome.nih.gov/) and GTEx were accessed through the UCSC Xena platform (https://xenabrowser.net).
