# Supplementary material for: Association between Vitamin B12 Levels and Colon Cancer Survival: A Global Network Study
Source: Cancer Res Commun. 2026 Feb 11;6(2):302–9. doi: 10.1158/2767-9764.CRC-25-0557 (PMC13134766; doi:10.1158/2767-9764.CRC-25-0557)
Supplement: Supplemental Figure S1 — Kaplan-Meier curves based on B12 status after propensity score matching. [file crc-25-0557_supplemental_figure_s1_suppsf1.docx]

**Supplemental Figure S1. Kaplan-Meier curves based on B12 status after propensity score matching.** Colon cancer patients were stratified into cohorts based on B12 levels and cohorts were matched based on demographics, medical conditions, medications, and procedures before survival analyses. **A.** High B12 levels are associated with decreased survival when compared to low B12 patients (n= 4,982 in both cohorts) (mOS 64.0 vs 142.9 months, p<0.001, HR= 1.98, 95% CI 1.84-2.13). **B.** High B12 levels are associated with decreased survival when compared to normal B12 patients (n= 6,436 in each cohort) (mOS 59.4 vs 117.8 months, p<0.001, HR= 1.65, 95% CI 1.55-1.75). **C.** Normal B12 was not associated with a difference in survival compared to low B12 patients (n=5,131 in each cohort) (mOS 132.1 vs 142.9 months, p=0.05, HR=1.10, 95% CI 0.99-1.20). Statistical significance was evaluated by log-rank test and Cox proportional hazards model with 95% confidence intervals.
