# Supplementary material for: Association between Vitamin B12 Levels and Colon Cancer Survival: A Global Network Study
Source: Cancer Res Commun. 2026 Feb 11;6(2):302–9. doi: 10.1158/2767-9764.CRC-25-0557 (PMC13134766; doi:10.1158/2767-9764.CRC-25-0557)
Supplement: Supplemental Figure S2 — Gene expression levels of enzymes involved in methionine and folate cycle and colon cancer survival. [file crc-25-0557_supplemental_figure_s2_suppsf2.docx]

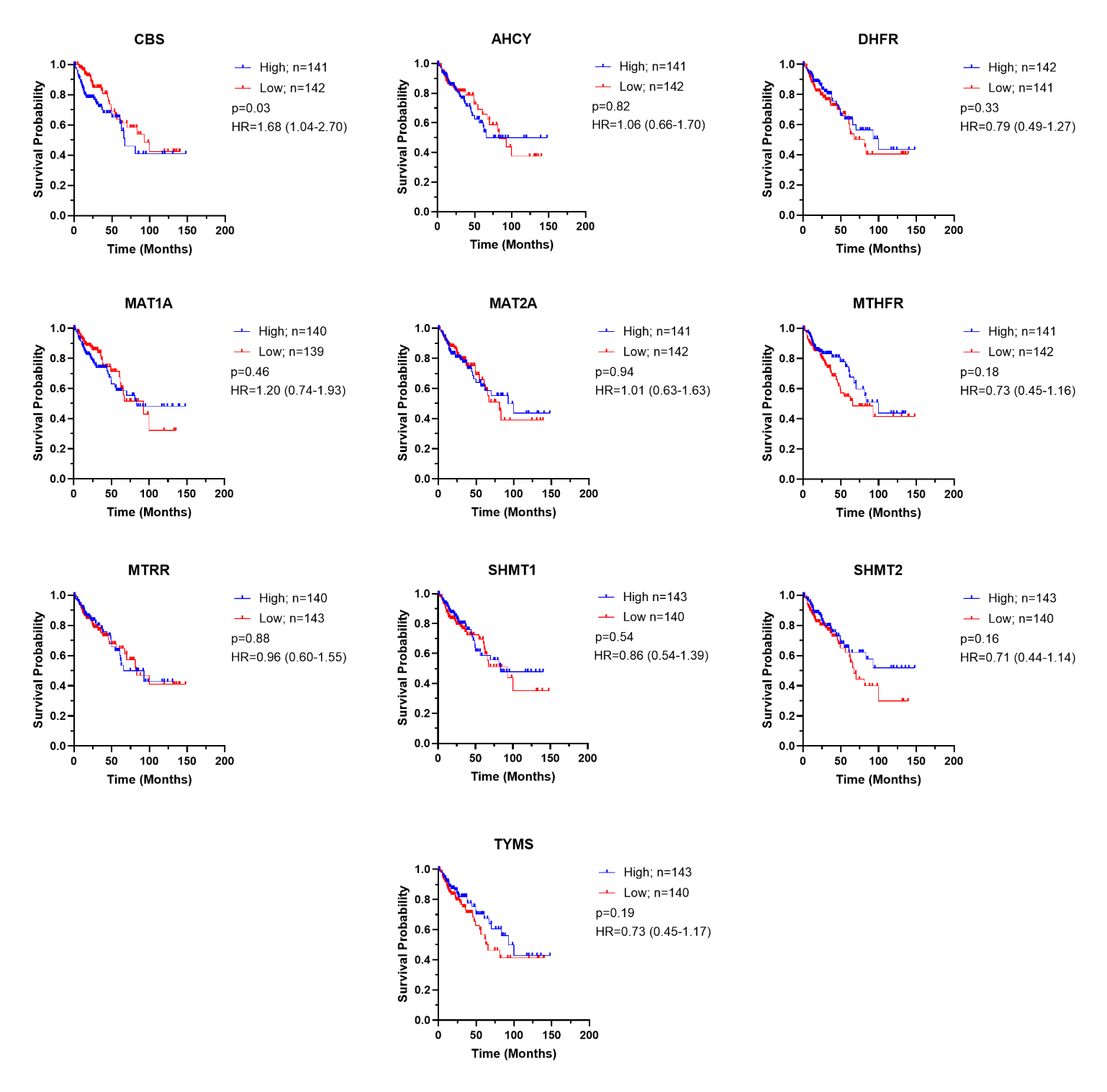


**Supplemental Figure S2. Gene expression levels of enzymes involved in methionine and folate cycle and colon cancer survival.** RNAseq data from TCGA was accessed through the UCSC Xena Browser platform. HR’s with 95% CI were determined using the Mantel-Haenszel model and statistical significance was determined by log-rank test.
