# Supplementary material for: Association between Vitamin B12 Levels and Colon Cancer Survival: A Global Network Study
Source: Cancer Res Commun. 2026 Feb 11;6(2):302–9. doi: 10.1158/2767-9764.CRC-25-0557 (PMC13134766; doi:10.1158/2767-9764.CRC-25-0557)
Supplement: Supplemental Table S1 — Characteristics of High and Low B12 Colon Cancer Patients Before and After Propensity Score Matching. [file crc-25-0557_supplemental_table_s1_suppst1.docx]

|  | **Before matching** | | | | **After matching** | | | |
| --- | --- | --- | --- | --- | --- | --- | --- | --- |
|  | High B12 n=6,523 | Low B12 n=5,167 | p-value^b^ | High B12 n=4,982 | | Low B12 n=4,982 | p-value^b^ |  |
| **Demographics** |  |  |  |  | |  |  |  |
| Age at Index, Mean (SD), y | 66.0 (12.5) | 65.0 (12.4) | <0.001 | 65.4 (12.5) | | 65.2 (12.4) | 0.45 |  |
| Male | 46.9% | 49.3% | 0.01 | 48.4% | | 48.7% | 0.76 |  |
| Female | 53.1% | 50.7% | 0.01 | 51.6% | | 51.3% | 0.76 |  |
| White | 68.2% | 76.6% | <0.001 | 76.0% | | 75.8% | 0.78 |  |
| Not Hispanic/Latino | 74.4% | 75.0% | 0.51 | 74.9% | | 74.6% | 0.76 |  |
| Hispanic/Latino | 6.3% | 5.0% | 0.002 | 4.9% | | 5.2% | 0.46 |  |
| Black/African American | 18.1% | 12.9% | <0.001 | 13.3% | | 13.4% | 0.91 |  |
| Asian | 4.8% | 2.2% | <0.001 | 2.3% | | 2.2% | 0.84 |  |
| **Medical Conditions** |  |  |  |  | |  |  |  |
| Delirium | 0.6% | 0.3% | 0.02 | 0.2% | | 0.3% | 0.24 |  |
| Neutropenia | 2.2% | 1.3% | <0.001 | 1.3% | | 1.3% | 0.93 |  |
| Pancytopenia | 2.4% | 1.1% | <0.001 | 1.0% | | 1.1% | 0.70 |  |
| **Metastatic Diagnosis** |  |  |  |  | |  |  |  |
| Lymph nodes | 3.2% | 2.1% | <0.001 | 2.0% | | 2.1% | 0.67 |  |
| Respiratory and digestive organs | 8.1% | 4.5% | <0.001 | 4.5% | | 4.7% | 0.63 |  |
| Unspecified | 4.9% | 3.1% | <0.001 | 3.1% | | 3.3% | 0.53 |  |
| **Procedures** |  |  |  |  | |  |  |  |
| Colonoscopy w/ removal (snare) | 3.7% | 4.2% | 0.16 | 3.6% | | 4.0% | 0.29 |  |
| Partial colectomy | 0.5% | 0.8% | 0.03 | 0.5% | | 0.6% | 0.51 |  |
| Partial colectomy w/ ileocolostomy | 0.3% | 0.5% | 0.08 | 0.3% | | 0.4% | 0.29 |  |
| **Medications** |  |  |  |  | |  |  |  |
| Radiation Therapy | 3.0% | 3.0% | 1.00 | 2.5% | | 2.7% | 0.57 |  |
| oxaliplatin | 2.4% | 2.1% | 0.32 | 1.9% | | 2.1% | 0.57 |  |
| fluorouracil | 2.8% | 2.8% | 1.00 | 2.4% | | 2.6% | 0.44 |  |
| capecitabine | 1.5% | 1.9% | 0.13 | 1.5% | | 1.4% | 0.74 |  |
| leucovorin | 2.1% | 2.0% | 0.66 | 1.8% | | 1.9% | 0.60 |  |
| B12 and folic acid | 12.3% | 7.8% | <0.001 | 7.9% | | 8.1% | 0.66 |  |
| **Laboratory** |  |  |  |  | |  |  |  |
| **Folate, Mean (SD), ng/mL** | 14.3 (18.6) | 12.2 (7.6) | 0.03 | 12.5 (6.4) | | 12.3 (7.6) | 0.57 |  |
| 0 - 10 ng/mL | 3.7% | 3.6% | 0.76 | 3.2% | | 3.7% | 0.19 |  |
| 10 - 20 ng/mL | 5.9% | 4.9% | 0.02 | 4.6% | | 5.0% | 0.30 |  |
| > 20 ng/mL | 1.0% | 0.8% | 0.17 | 0.7% | | 0.8% | 0.41 |  |

**Supplemental Table S1. Characteristics of High and Low B12 Colon Cancer Patients Before and After Propensity Score Matching^a^.** ^a^Colon cancer patient cohorts were determined by measured B12 levels within 1 y after initial colon cancer diagnosis. Patients with High B12 were defined as having serum/plasma values measured at > 1000 pg/mL and Low B12 was defined as < 300 pg/mL.

^b^Test for significant difference between cohorts based on TriNetX Analytics.
