# Supplementary material for: Association between Vitamin B12 Levels and Colon Cancer Survival: A Global Network Study
Source: Cancer Res Commun. 2026 Feb 11;6(2):302–9. doi: 10.1158/2767-9764.CRC-25-0557 (PMC13134766; doi:10.1158/2767-9764.CRC-25-0557)
Supplement: Supplemental Table S2 — Characteristics of High and Normal B12 Colon Cancer Patients Before and After Propensity Score Matching. [file crc-25-0557_supplemental_table_s2_suppst2.docx]

|  | **Before matching** | | | **After matching** | | |
| --- | --- | --- | --- | --- | --- | --- |
|  | High B12 n=6,523 | Normal B12  n=23,965 | p-value^b^ | High B12 n=6,436 | Normal B12 n=6,436 | p-value^b^ |
| **Demographics** |  |  |  |  |  |  |
| Age at Index, Mean (SD), y | 66.0 (12.5) | 64.8 (12.7) | <0.001 | 66.0 (12.4) | 66.1 (12.4) | 0.69 |
| Male | 46.8% | 50.4% | <0.001 | 46.9% | 46.8% | 0.96 |
| Female | 53.1% | 49.6% | <0.001 | 53.1% | 53.1% | 0.96 |
| White | 68.1% | 72.5% | <0.001 | 68.2% | 68.2% | 0.93 |
| Not Hispanic/Latino | 74.3% | 73.1% | 0.04 | 74.3% | 74.7% | 0.62 |
| Hispanic/Latino | 6.3% | 5.3% | <0.001 | 6.3% | 6.4% | 0.83 |
| Black/African American | 18.2% | 14.8% | <0.001 | 18.2% | 18.4% | 0.75 |
| Asian | 4.8% | 3.4% | <0.001 | 4.7% | 4.6% | 0.77 |
| **Medical Conditions** |  |  |  |  |  |  |
| Delirium | 0.6% | 0.5% | 0.29 | 0.6% | 0.5% | 0.48 |
| Neutropenia | 2.3% | 1.7% | 0.001 | 2.2% | 1.7% | 0.06 |
| Pancytopenia | 2.4% | 1.4% | <0.001 | 2.2% | 1.8% | 0.08 |
| **Metastatic Diagnosis** |  |  |  |  |  |  |
| Lymph nodes | 3.1% | 2.3% | <0.001 | 3.1% | 2.9% | 0.54 |
| Respiratory/digestive organs | 8.1% | 4.8% | <0.001 | 7.9% | 7.8% | 0.70 |
| Unspecified | 4.9% | 2.8% | <0.001 | 4.8% | 4.5% | 0.46 |
| **Procedures** |  |  |  |  |  |  |
| Colonoscopy w/ removal (snare) | 3.7% | 4.2% | 0.08 | 3.7% | 3.6% | 0.82 |
| Partial colectomy | 0.5% | 0.6% | 0.55 | 0.5% | 0.4% | 0.29 |
| Partial colectomy w/ ileocolostomy | 0.3% | 0.2% | 0.04 | 0.3% | 0.3% | 0.52 |
| **Medications** |  |  |  |  |  |  |
| Radiation therapy | 3.1% | 2.6% | 0.02 | 3.0% | 2.5% | 0.05 |
| oxaliplatin | 2.4% | 2.1% | 0.11 | 2.4% | 2.1% | 0.21 |
| fluorouracil | 2.8% | 2.9% | 0.74 | 2.8% | 2.4% | 0.23 |
| capecitabine | 1.5% | 1.4% | 0.59 | 1.5% | 1.1% | 0.05 |
| leucovorin | 2.1% | 1.9% | 0.37 | 2.1% | 1.8% | 0.23 |
| B12 and folic acid | 12.4% | 8.6% | <0.001 | 12.4% | 12.5% | 0.77 |
| **Laboratory** |  |  |  |  |  |  |
| **Folate, Mean (SD), ng/mL** | 14.2 (18.6) | 13.1 (13) | 0.07 | 14.2 (18.7) | 14.1 (17.7) | 0.85 |
| 0 - 10 ng/mL | 3.7% | 3.4% | 0.23 | 3.7% | 3.1% | 0.07 |
| 10 - 20 ng/mL | 5.9% | 5.3% | 0.05 | 5.9% | 5.5% | 0.31 |
| > 20 ng/mL | 1.0% | 0.8% | 0.14 | 1.0% | 1.0% | 0.79 |

**Supplemental Table S2. Characteristics of High and Normal B12 Colon Cancer Patients Before and After Propensity Score Matching^a^.** ^a^Colon cancer patient cohorts were determined by measured B12 levels within 1 y after initial colon cancer diagnosis. Patients with High B12 were defined as having serum/plasma values measured at > 1000 pg/mL and Normal B12 was defined as 300-1000 pg/mL.

^b^Test for significant difference between cohorts based on TriNetX Analytics.
