# Supplementary material for: Association between Vitamin B12 Levels and Colon Cancer Survival: A Global Network Study
Source: Cancer Res Commun. 2026 Feb 11;6(2):302–9. doi: 10.1158/2767-9764.CRC-25-0557 (PMC13134766; doi:10.1158/2767-9764.CRC-25-0557)
Supplement: Supplemental Table S3 — Characteristics of Normal and Low B12 Colon Cancer Patients Before and After Propensity Score Matching. [file crc-25-0557_supplemental_table_s3_suppst3.docx]

|  | **Before matching** | | | **After matching** | | |
| --- | --- | --- | --- | --- | --- | --- |
|  | Normal B12 n=23,965 | Low B12  n= 5,167 | p-value^b^ | Normal B12 n=5,131 | Low B12 n=5,131 | p-value^b^ |
| **Demographics** |  |  |  |  |  |  |
| Age at Index, Mean (SD), y | 64.9 (12.7) | 65.0 (12.4) | 0.51 | 65.1 (12.5) | 65.0 (12.4) | 0.76 |
| Male | 50.4% | 49.3% | 0.15 | 49.5% | 49.3% | 0.84 |
| Female | 49.6% | 50.7% | 0.15 | 50.5% | 50.7% | 0.84 |
| White | 72.5% | 76.6% | <0.001 | 76.2% | 76.6% | 0.68 |
| Not Hispanic/Latino | 73.2% | 75.0% | 0.01 | 74.3% | 74.9% | 0.50 |
| Hispanic/Latino | 5.3% | 5.0% | 0.39 | 4.7% | 5.0% | 0.44 |
| Black/African American | 14.8% | 12.9% | <0.001 | 13.0% | 12.9% | 0.93 |
| Asian | 3.3% | 2.2% | <0.001 | 2.1% | 2.2% | 0.73 |
| **Medical Conditions** |  |  |  |  |  |  |
| Delirium | 0.5% | 0.3% | 0.07 | 0.2% | 0.3% | 0.32 |
| Neutropenia | 1.7% | 1.3% | 0.06 | 0.8% | 1.3% | 0.03 |
| Pancytopenia | 1.4% | 1.1% | 0.08 | 0.9% | 1.0% | 0.42 |
| **Metastatic Diagnosis** |  |  |  |  |  |  |
| Lymph nodes | 2.3% | 2.1% | 0.29 | 1.7% | 2.1% | 0.11 |
| Respiratory/digestive organs | 4.8% | 4.5% | 0.38 | 3.8% | 4.5% | 0.07 |
| Unspecified | 2.8% | 3.1% | 0.17 | 2.7% | 3.1% | 0.16 |
| **Procedures** |  |  |  |  |  |  |
| Colonoscopy w/ removal (snare) | 4.2% | 4.2% | 0.93 | 3.8% | 4.2% | 0.37 |
| Partial colectomy | 0.5% | 0.8% | 0.03 | 0.6% | 0.7% | 0.47 |
| Partial colectomy w/ ileocolostomy | 0.2% | 0.5% | <0.001 | 0.3% | 0.3% | 0.73 |
| **Medications** |  |  |  |  |  |  |
| Radiation therapy | 2.5% | 3.0% | 0.04 | 2.3% | 3.0% | 0.02 |
| oxaliplatin | 2.1% | 2.1% | 0.80 | 1.6% | 2.1% | 0.06 |
| fluorouracil | 2.8% | 2.8% | 0.79 | 1.9% | 2.8% | 0.01 |
| capecitabine | 1.4% | 1.9% | 0.01 | 1.6% | 1.8% | 0.29 |
| leucovorin | 1.9% | 2.0% | 0.75 | 1.4% | 2.0% | 0.02 |
| B12 and folic acid | 8.6% | 7.8% | 0.09 | 6.9% | 7.8% | 0.08 |
| **Laboratory** |  |  |  |  |  |  |
| **Folate, Mean (SD), ng/mL** | 13.1 (13.0) | 12.2 (7.6) | 0.15 | 14.0 (20.6) | 12.2 (7.6) | 0.08 |
| 0 - 10 ng/mL | 3.4% | 3.6% | 0.49 | 3.1% | 3.6% | 0.19 |
| 10 - 20 ng/mL | 5.3% | 4.9% | 0.25 | 4.6% | 4.8% | 0.64 |
| > 20 ng/mL | 0.8% | 0.8% | 0.69 | 0.6% | 0.8% | 0.34 |

**Supplemental Table S3. Characteristics of Normal and Low B12 Colon Cancer Patients Before and After Propensity Score Matching^a^.** ^a^Colon cancer patient cohorts were determined by measured B12 levels within 1 y after initial colon cancer diagnosis. Patients with Low B12 were defined as having serum/plasma values measured at < 300 pg/mL and Normal B12 was defined as 300-1000 pg/mL.

^b^Test for significant difference between cohorts based on TriNetX Analytics.
