# Supplementary material for: Association between Vitamin B12 Levels and Colon Cancer Survival: A Global Network Study
Source: Cancer Res Commun. 2026 Feb 11;6(2):302–9. doi: 10.1158/2767-9764.CRC-25-0557 (PMC13134766; doi:10.1158/2767-9764.CRC-25-0557)
Supplement: Supplemental Table S4 — Background characteristics of maladaptive B12 conditions across cohorts. [file crc-25-0557_supplemental_table_s4_suppst4.docx]

|  | **High B12** | **Normal B12** | **p-value^a^ (High vs Normal)** | **Low B12** | **p-value**  **(High vs Low)** |
| --- | --- | --- | --- | --- | --- |
| **Procedures^b^** |  |  |  |  |  |
| Total gastrectomy | 0% | **≤0.1**% | **–** | **≤0.2**% | **–** |
| Partial gastrectomy | **≤0.2**% | **≤0.1**% | **–** | **≤0.2**% | **–** |
| Bariatric surgery | **≤0.2**% | **≤0.1**% | **–** | **≤0.2**% | **–** |
| **Medical conditions** |  |  |  |  |  |
| Pernicious anemia | 0.4% | 0.3% | 0.56 | **≤0.2**% | **–** |
| Gastritis and duodenitis | 3.6% | 3.7% | 0.97 | 3.4% | 0.54 |
| Crohn’s disease | 1.1% | 1.2% | 0.51 | 1.3% | 0.26 |
| Celiac disease | 0.2% | 0.2% | 0.51 | **≤0.2**% | **–** |

**Supplemental Table S4.** Background characteristics of maladaptive B12 conditions across cohorts. ^a^p-values were omitted for comparisons involving rare characteristics with suppressed patient counts (≤10) for privacy. ^b^Procedures were determined by at least one recorded procedure within 1 y prior to index event.
