# Supplementary material for: Association between Vitamin B12 Levels and Colon Cancer Survival: A Global Network Study
Source: Cancer Res Commun. 2026 Feb 11;6(2):302–9. doi: 10.1158/2767-9764.CRC-25-0557 (PMC13134766; doi:10.1158/2767-9764.CRC-25-0557)
Supplement: Supplemental Table S5 — Cancer staging data from colon cancer patients at index event with high, normal, and low B12 values. [file crc-25-0557_supplemental_table_s5_suppst5.docx]

|  | High B12 (n= 355) | Normal B12  (n= 1,175) | p-value  (High vs Normal) | Low B12  (n=185) | p-value  (High vs Low) |
| --- | --- | --- | --- | --- | --- |
| **TNM Staging** |  |  |  |  |  |
| **Tumor** |  |  |  |  |  |
| T1 | 11% | 11% | 0.92 | 12% | 0.68 |
| T2 | 6% | 9% | 0.20 | 14% | 0.004 |
| T3 | 30% | 41% | <0.001 | 42% | 0.01 |
| T4 | 32% | 26% | 0.049 | 23% | 0.04 |
| **Node** |  |  |  |  |  |
| N0 | 57% | 63% | 0.05 | 64% | 0.14 |
| N1 | 32% | 31% | 0.75 | 30% | 0.66 |
| N2 | 13% | 16% | 0.15 | 17% | 0.21 |
| N3 | - | - | - | - | - |
| **Metastasis** |  |  |  |  |  |
| M0 | 46% | 63% | <0.001 | 53% | <0.001 |
| M1 | 53% | 35% | <0.001 | 26% | <0.001 |
| **Tumor Staging** |  |  |  |  |  |
| Stage 1 | 12% | 16% | <0.001 | 20% | 0.01 |
| Stage 2 | 15% | 25% | <0.001 | 24% | 0.01 |
| Stage 3 | 23% | 29% | 0.03 | 31% | 0.04 |
| Stage 4 | 54% | 36% | <0.001 | 28% | <0.001 |

**Supplemental Table S5.** Cancer staging data from colon cancer patients at index event with high, normal, and low B12 values.
